# Bridging disciplines to advance elasmobranch conservation: applications of physiological ecology

**DOI:** 10.1093/conphys/coz011

**Published:** 2019-05-15

**Authors:** K Lyons, J S Bigman, D Kacev, C G Mull, A B Carlisle, J L Imhoff, J M Anderson, K C Weng, A S Galloway, E Cave, T R Gunn, C G Lowe, R W Brill, C N Bedore

**Affiliations:** 1 Georgia Aquarium, Atlanta, GA, USA; 2 Simon Fraser University, Burnaby, Canada; 3 Southwest Fisheries Science Center, La Jolla, CA, USA; 4 University of Delaware, DE, USA; 5 Florida State University Coastal and Marine Laboratory, St. Teresa, FL, USA; 6 University of Hawai`i at Mānoa, Honolulu, HI, USA; 7 Virginia Institute of Marine Science, Gloucester Point, VA, USA; 8 South Carolina Department of Natural Resources, SC, USA; 9 Florida Atlantic University, Boca Raton, FL, USA; 10 Georgia Southern University, Statesboro, GA USA; 11 California State University Long Beach, Long Beach, CA, USA

**Keywords:** Conservation, elasmobranch, physiological ecology

## Abstract

A strength of physiological ecology is its incorporation of aspects of both species’ ecology and physiology; this holistic approach is needed to address current and future anthropogenic stressors affecting elasmobranch fishes that range from overexploitation to the effects of climate change. For example, physiology is one of several key determinants of an organism’s ecological niche (along with evolutionary constraints and ecological interactions). The fundamental role of physiology in niche determination led to the development of the field of physiological ecology. This approach considers physiological mechanisms in the context of the environment to understand mechanistic variations that beget ecological trends. Physiological ecology, as an integrative discipline, has recently experienced a resurgence with respect to conservation applications, largely in conjunction with technological advances that extended physiological work from the lab into the natural world. This is of critical importance for species such as elasmobranchs (sharks, skates and rays), which are an especially understudied and threatened group of vertebrates. In 2017, at the American Elasmobranch Society meeting in Austin, Texas, the symposium entitled `Applications of Physiological Ecology in Elasmobranch Research’ provided a platform for researchers to showcase work in which ecological questions were examined through a physiological lens. Here, we highlight the research presented at this symposium, which emphasized the strength of linking physiological tools with ecological questions. We also demonstrate the applicability of using physiological ecology research as a method to approach conservation issues, and advocate for a more available framework whereby results are more easily accessible for their implementation into management practices.

## Teaser

Physiology and ecology are intimately linked; thus, ecological observations can be strengthened by an understanding of organismal physiology. Through a symposium entitled “Applications of Physiological Ecology in Elasmobranch Research” at the 2017 American Elasmobranch Society meeting, we demonstrated the strengths of collaborative efforts across disciplines for informing conservation efforts.

## Introduction

Physiology has traditionally been studied from the biochemical to organismal level, without consideration for the effects of or interaction with aspects of the organism’s habitat, which include both abiotic and biotic factors. In this limited scope, our understanding of physiological mechanisms does not account for physiological variability that is attributable to ecological factors when extrapolating laboratory experiments to field settings. Likewise, ecological studies cannot fully explain patterns of animal behaviour without considering the underlying physiological mechanism(s) that influence those observations. Physiological ecology integrates the physiology of organisms within the context of their environment and evolutionary histories (McNab, 2002); thus, it bridges this gap between disciplines and enhances conservation efforts by improving our understanding of how physiology influences the distribution and ecology of organisms and our ability to predict the ways in which animals may respond to changes in their environment.

The field of physiological ecology has experienced a recent resurgence as advances in technology (e.g. accelerometry, computing power to handle `big data’) expand the capability to incorporate physiological measurements into ecological studies that address conservation concerns in species of interest (Helmuth *et al.*, 2004; Kearney and Porter, 2009). Because of these technological advancements, physiological ecology studies are no longer restricted to the laboratory, and consequently the number and types of taxa studied has increased. This is especially true for elasmobranch fishes (sharks, skates and rays), as much of this group is difficult to study due to their large size, high mobility or the difficulty of being maintained in captivity (Lowe and Goldman, 2001; Bernal *et al.*, 2012; Bernal and Lowe, 2015). As such, traditional laboratory research tends to focus on small, sedentary species (e.g. species referenced in Ballantyne, 1997). These challenges have historically limited the scope of research in elasmobranchs, leading to a lag in our understanding of their biology relative to other vertebrates. Approaching research questions from a physiological ecological framework, including the application of recent technological advances—such as those outlined in this *Perspective*—has the potential to improve our understanding of the intricate relationship between physiology and ecology in elasmobranch fishes and how it can be applied to conservation and management.

**Figure 1:**
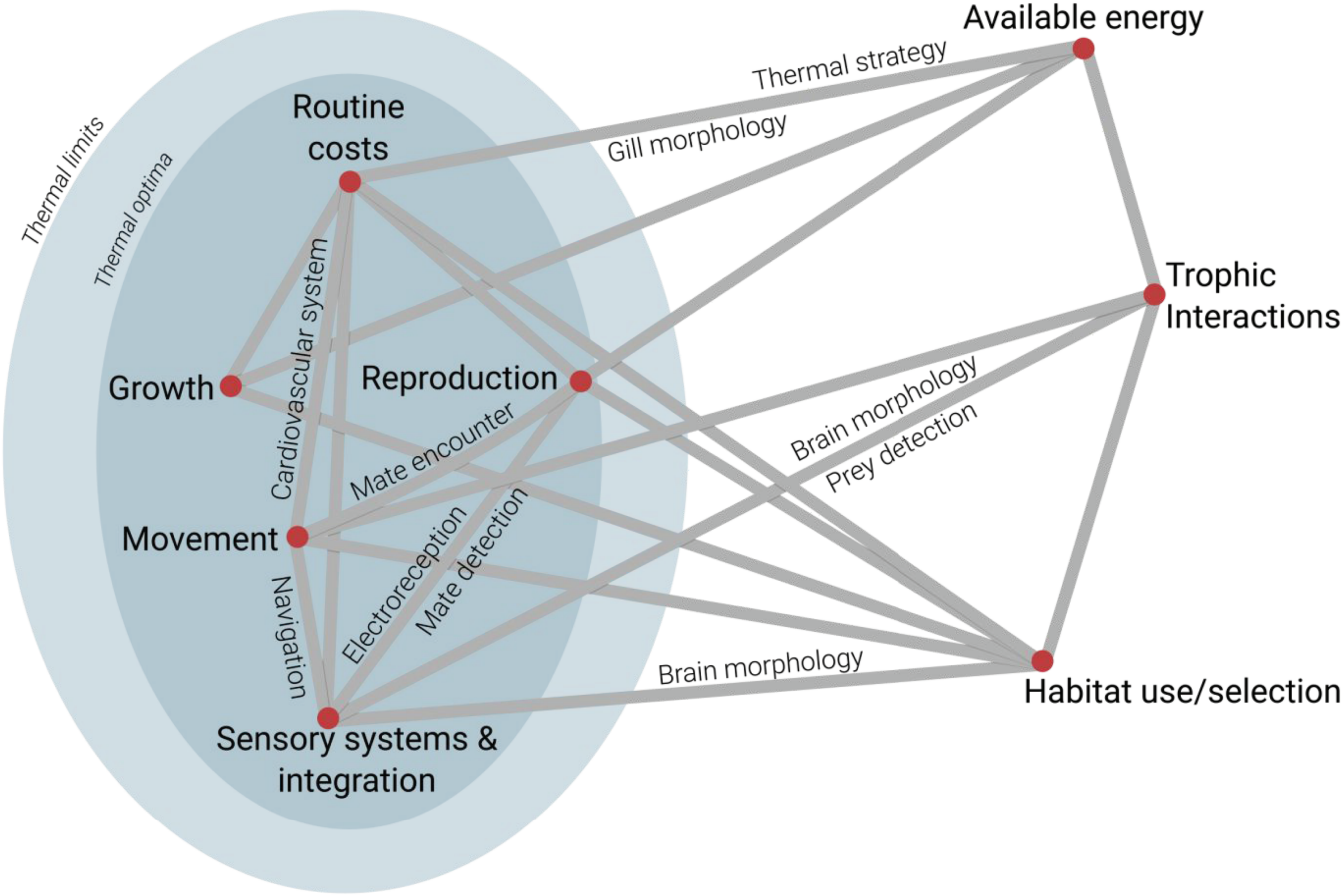
A species’ physiology often underpins many traditional `ecological characteristics’. For example, many processes are thermally constrained (e.g. illustrated by blue ellipses). Thus, both ecological and physiological factors influence how an animal interacts with its environment. Here, we highlight symposium concepts presented and discussed at the 2017 American Elasmobranch Society annual meeting. This figure highlights some of the complex interactions of ecology and physiology, but is not inclusive of every interaction for every elasmobranch species. Rather, we use this to illustrate how the field of physiological ecology can be used to address elasmobranch conservation issues as it holistically accounts for aspects of species’ biology.

Physiological ecology has the potential to play an important role in conservation by furthering our understanding of the connection between underlying physiological mechanisms and ecological observations and patterns (Fig. 1). Compared to other marine vertebrate taxa, elasmobranchs are one of the most intrinsically sensitive groups to extinction, yet understudied; one quarter of all species are threatened with a high risk of extinction and almost one-half are unable to be assessed due to the lack of data (Dulvy *et al.*, 2008, 2014; McClenachan *et al.*, 2011). While overexploitation is the primary driver of elevated extinction risk (Dulvy *et al.*, 2014), other anthropogenic influences potentially threaten elasmobranchs such as habitat destruction (Jennings *et al.*, 2008), marine pollution (Lyons and Wynne-Edwards, 2018) and climate change (Rosa *et al.*, 2014; Pistevos *et al.*, 2015). Integrating physiological mechanisms into ecological studies has the potential to improve our understanding of ecological phenomena, and ultimately, may help to mitigate these threats by tailoring conservation strategies and efforts. For example, understanding how thermal preferences and tolerance affect the range and distribution is important when considering management measures such as regional fishery closures. Additionally, marine pollution studies might help us understand the effects of a specific substance on a species reproductive output, knowledge that would be important for initiating bans on that substance. Employing physiological techniques outside the laboratory and broadening our focus to species that have been difficult to study using traditional approaches also has potential in improving targeted efforts. For example, measuring metabolic rate on species that cannot easily be brought into the laboratory would improve our understanding of their life history traits, which are directly used in stock assessments to manage fisheries. However, integrating physiological mechanisms into ecological studies is not enough; the challenge remains to scale up these individual studies on a scale that is relevant to policy makers and stakeholders (Cooke *et al.*, 2014). For example, empirically linking metabolic rate to life history traits such as growth is needed before metabolic rate has utility in being applied to management and conservation. Collaboration between ecologists and physiologists is essential to reaching the full potential of incorporating physiology into ecological studies, as well as applying these conclusions to inform conservation and management efforts.

**Figure 2:**
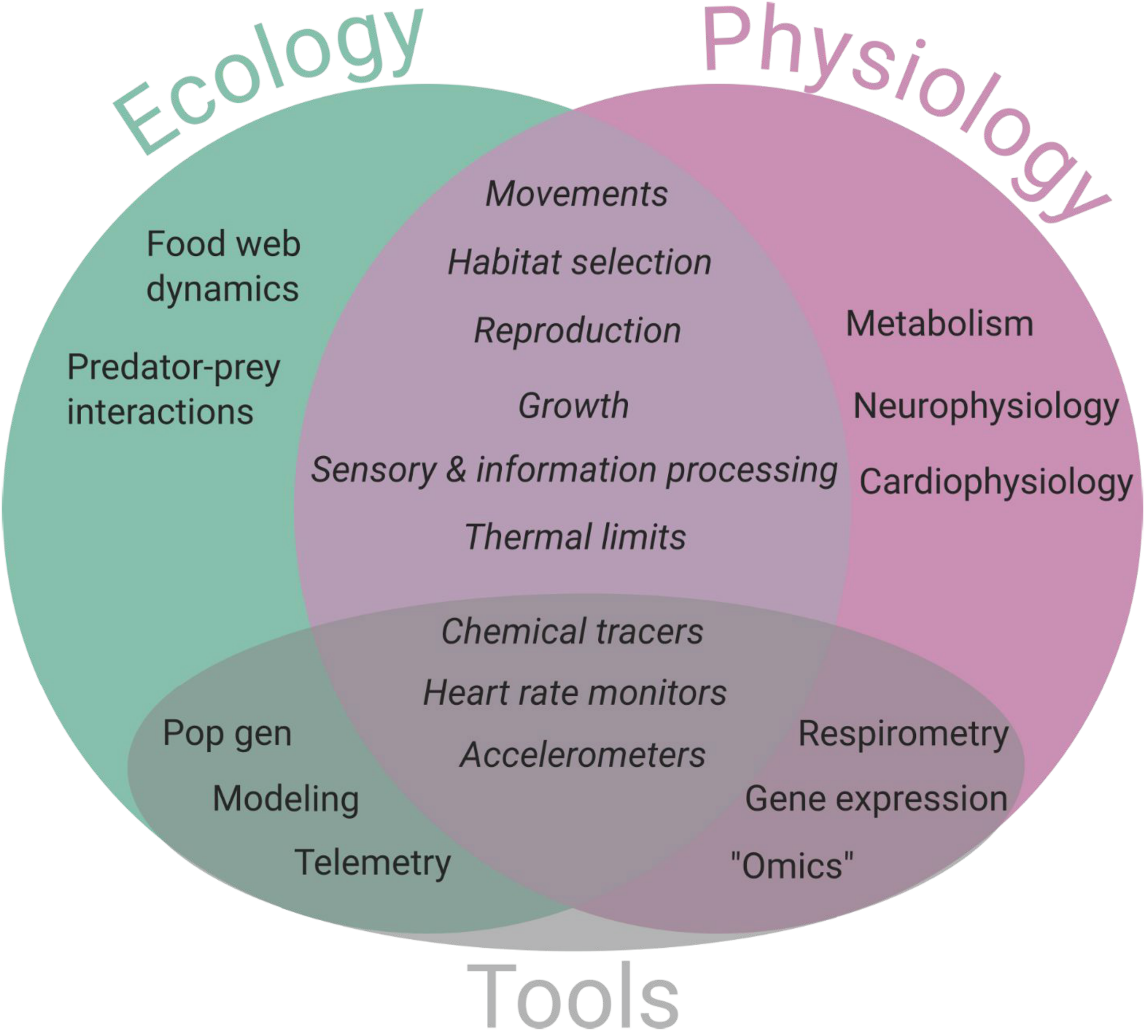
The disciplines of ecology (green) and physiology (purple) are traditionally viewed as distinct areas of study; however, more often studies are integrating information from both of these fields, as demonstrated by the overlap. At the American Elasmobranch Society annual meeting, our symposium highlighted research at the intersection of these disciplines along with current tools being used to address elasmobranch conservation challenges.

The application of new methods and technologies to elasmobranch research naturally leads to increased linkages between disciplines as both ecological and physiological perspectives are needed to interpret data in meaningful, biologically relevant ways (Feder and Block, 1991; Cooke *et al.*, 2008; Baktoft *et al.*, 2016; Fig. 2). However, despite working towards a common conservation goal, elasmobranch physiologists and ecologists tend to answer research questions in silos according to their traditional disciplines. At the 2017 American Elasmobranch Society annual meeting in Austin, TX, we hosted a symposium entitled `Applications of Physiological Ecology to Elasmobranch Research’ to encourage interdisciplinary collaboration for promoting elasmobranch conservation. In this *Perspective*, we summarize key concepts and tools presented by the speakers that demonstrate the need to examine results from a physiological ecology perspective and how doing so can potentially further conservation and management applications. We outline the state of knowledge in the fields of (i) metabolism and energy use, (ii) thermal physiology, (iii) sensory ecology and neuroecology, providing a broad overview of available methods and tools and outline key future directions and questions for the field of elasmobranch physiological ecology. While we recognize this *Perspective* cannot encompass all applications of physiological ecology research, we hope it can foster the development of this field to address the sensitive conservation needs of many elasmobranch species.

### Metabolism and energy use

Aerobic scope—the difference between maximum aerobic metabolic rate and standard metabolic rate (i.e. minimum metabolic rate)—forms a dynamic connection between ecology and physiology, as it governs the rate at which organisms assimilate resources from the environment into available energy for metabolism, growth and reproduction (Priede, 1985; Brown *et al.*, 2004; Careau *et al.*, 2014). Thus, metabolic rate in general (i.e. all types of aerobic metabolic rate and for simplicity, hereafter, `metabolic rate’ unless otherwise specified) underpins life histories and has been linked empirically to both individual and population growth rates (Hennemann, 1983; Pettersen *et al.*, 2015, 2016). As population growth rates are a correlate of extinction risk and potential resilience to fishing pressure, metabolic rate has the potential to effectively bridge the fields of physiology, ecology and conservation.

Metabolic rates are not well-documented for elasmobranch fishes; estimates of metabolic rate, whether standard or routine (see Careau *et al.*, 2014 for specific definitions) are only published for 24 species (or 0.02% of all species; Hughes, 1978; Carlson *et al.*, 2004; Bernal *et al.*, 2012; Bernal and Lowe, 2015) and measurement of maximum metabolic rates for even fewer (Brill and Lai, 2016). Additionally, these data are for mostly small-bodied and/or less active species (Lowe and Goldman, 2001; Carlson *et al.*, 2004; Bernal and Lowe, 2015) that can be maintained in captivity; thus, the utility of using metabolic rate to enhance our understanding of ecological phenomena has been hindered by the logistical difficulties in quantifying this trait through traditional means (e.g. respirometry and swim tunnels). Advances in technology have made estimating metabolic rate feasible for both larger-bodied and more active species using physiological telemetry and accelerometry (Lowe and Goldman, 2001; Carlson *et al.*, 2004; Bernal *et al.*, 2012; Bernal and Lowe, 2015). Physiological telemetry entails correlation of physiological parameters (e.g. heart rate, swimming speed, muscle contraction rate, tail beat frequency or overall body activity) with behaviour (e.g. activity levels, habitat use and migratory patterns; Lucas *et al.*, 1993). Accelerometry, as a tool to estimate energy expenditure in the field, has enabled metabolic rate to be estimated for a variety of traditionally difficult-to-study elasmobranchs (Gleiss *et al.*, 2010; Barnett *et al.*, 2016; Lear *et al.*, 2017). For example, Lear *et al.* (2017) used accelerometry to estimate the metabolic rate of free-ranging nurse sharks (*Ginglymostoma cirratum*), lemon sharks (*Negaprion brevirostris*) and blacktip sharks (*Carcharhinus limbatus*) in conjunction with laboratory calibrations. Metabolic rate for these species can now be predicted using accelerometry in the field (Lear *et al.*, 2017). Accelerometry has also facilitated the integration of ecological processes (e.g. influences from abiotic and biotic factors) within the scope of applied physiology (Whitney *et al.*, 2007; Gleiss *et al.*, 2009; Lear *et al.*, 2017).

Other approaches to understanding energy expenditure and availability include the use of modelling to identify correlates of metabolic rate (Sims, 2000; Gillooly *et al.*, 2016; Bigman *et al*. (2017), ``Bigman unpublished data''). For example, gill morphology offers a proxy measurement for estimating metabolic rate, as the flux of oxygen across the gills is dependent on their surface area, with increasing surface area enhancing rates of oxygen uptake (Wegner, 2016). Both intraspecifically within and interspecifically across species, the relationship between gill surface area and mass is similar to that of maximum metabolic rate and mass, suggesting that gill surface area is matched to metabolic demand (Gillooly *et al.*, 2016; Wegner, 2016). Further, gill surface area and metabolic rate have a basis in ecology since both are correlated with temperature, activity and habitat type (Bernal *et al.*, 2012; Wootton *et al.*, 2015; Bigman *et al.*, 2018).

Heart rate is also strongly linked to metabolic rate, as well as maximum age (Hulbert *et al.*, 2007). Therefore, measurement of heart rate can provide insight into metabolic rate, energy expenditure and key determinants of resilience to exploitation (Farrell *et al.*, 2009; Cooke *et al.*, 2016). Whereas heart rate measurements for elasmobranchs have in the past required controlled laboratory settings (Chin Lai *et al.*, 1990), now there are novel methods of measuring heart rate in fish that can be used in mesocosm or even wild settings (Prystay *et al.*, 2017). Ongoing work seeks to apply these novel techniques in elasmobranchs.

### Thermal physiology

Of the various environmental factors that influence organismal physiology, few play as important a role as ambient temperature in shaping the ecology of ectothermic species. Ambient temperature influences the structure of cellular constituents and drives biochemical and metabolic rates (Fry, 1947; Somero *et al.*, 2017). Temperature, therefore, plays a role in growth and reproduction, as well as locomotor, cardiac, sensory and digestive performance (Bernal *et al.*, 2005; Donley *et al.*, 2007; Farrell *et al.*, 2009; Secor, 2009). Some elasmobranchs have been shown to exhibit ‘behavioral thermoregulation’, whereby individuals can alter their metabolic rate by traversing thermal gradients. This may optimize energy expenditure and physiological processes during feeding and reproduction (Carey *et al.*, 1990; Matern *et al.*, 2000; Hight and Lowe, 2007). The ability to modulate metabolic rate (and other physiological processes) through behaviour plays a fundamental role in structuring patterns of habitat use.

Regionally endothermic species (e.g. lamnid sharks) are unique among elasmobranch fishes for their ability to retain metabolically generated heat through the presence of counter-current heat exchangers, or *retia mirabilia*. This adaptation allows them to elevate the temperature of their red musculature above ambient by as much as 21°C in salmon sharks (*Lamna ditropis*; Carey *et al.*, 1985; Goldman *et al.*, 2004). Regional endothermy is also accompanied by a multitude of hypothesized physiological benefits (Block and Finnerty, 1994; Graham and Dickson, 2001; Watanabe *et al.*, 2015), such as enhanced sensory performance (Block and Carey, 1985; Tubbesing and Block, 2000). Furthermore, salmon sharks, and potentially other lamnids, also have specialized cardiac physiology that allows their heart to function effectively even at very low temperatures (Weng *et al.*, 2005). These anatomical and physiological adaptations allow regionally endothermic elasmobranchs to utilize wider niche spaces across broad thermal gradients and expand their range into cooler, often higher latitude ecosystems that tend to be more productive (Block and Finnerty, 1994; Graham and Dickson, 2001; Madigan *et al.*, 2015).

Thermal physiology plays a fundamental role in the ecology and biogeography in both strictly ectothermic and regionally endothermic elasmobranch fishes (Lowe and Goldman, 2001; Bernal *et al.*, 2012). Advances in electronic tagging approaches have increased the ability of researchers to record the stomach and muscle temperature *in situ* (Bernal *et al.*, 2012; Jorgensen *et al.*, 2015), providing new insights into the thermal ecology of these species. Understanding the thermal optima, physiological limits and other constraints of elasmobranch fishes provides a mechanistic understanding of how environmental conditions structure the distribution and ecology of these species. This type of information can be used to parameterize species-specific distribution and mechanistic niche models, which can be of great value to conservation and management, as they provide insights as to how the distribution and performance of a species might change as the oceans warm, deoxygenate and acidify under the influence of climate change (Pistevos *et al.*, 2015; Di Santo, 2016).

### Sensory ecology

Sensory systems are critical for animals to acquire information about their surrounding environments, and these systems must be optimized for their environmental conditions and behavioral requirements. Sensory systems not only allow animals to detect environmental conditions, but also enable prey detection even when prey are cryptic or found in low visibility habitats (Bedore *et al.*, 2015). The ability to forage effectively depends on the tuning of sensory function to environmental conditions (e.g. Bedore *et al.*, 2014).

Sensitivity and resolution are fundamental properties of all sensory systems and can give insight into the ecological needs of a species. For example, lemon sharks undergo an ontogenetic shift with respect to colour sensitivity as they move from green, estuarine nursery areas to blue, clearer water as subadults (Cohen *et al*. 1977), as do teleost species (Taylor *et al.*, 2011, 2015). Since species and life stages are uniquely adapted to particular environments, a comparative approach to sensory physiology is most informative relative to the ecological significance of these adaptations. Likewise, traditional ecological studies, such as identification of a species’ trophic niche, could benefit from incorporating sensory physiology, as prey detection is the foundation of successful foraging. Although sensitivity of the olfactory and electrosensory systems to chemical and electrical stimuli have been described (Kajiura and Holland, 2002; Meredith and Kajiura, 2010; Bedore *et al.*, 2013), data are lacking on cues used to identify and discriminate prey type (e.g. size, species, etc.). Knowledge of prey stimulus characteristics and responses to those stimuli (e.g. Bedore and Kajiura, 2013 and Bedore *et al.*, 2014) may lead to the implementation of effective barriers or deterrents to control elasmobranch access to baited hooks or areas of intensive aquaculture (Jordan *et al.*, 2013). Likewise, understanding how anthropogenic influences affect species’ sensory biology can aid conservation efforts by including how elasmobranchs’ ability to detect and capture prey may be affected by human-induced changes such as ocean acidification (Dixson *et al.*, 2014).

Sensory biology also plays a crucial role in the conservation and management of elasmobranchs, particularly with respect to reproduction. Identifying the physiological mechanisms enabling mature males and females to find each other at the right place and right time should be a high priority. For example, the electrosensory system has been implicated in mate detection and identification in stingrays (Tricas *et al.*, 1995; Sisneros and Tricas, 2000). Seasonal changes in circulating sex hormones (e.g. testosterone) of male Atlantic Stingrays (*Hypanus sabinus*) shifts sensitivity to that of conspecifics during the mating season, whereas decreasing androgen concentrations at the end of the mating season shifts sensitivity towards that of prey items (Sisneros and Tricas, 2000). Further investigation of the role of electroreception and other senses in mate detection, identification and selection in a wider range of elasmobranch species is needed to identify environmental conditions suitable for successful reproduction. This is especially significant considering that the maintenance or recovery of many elasmobranch populations is dependent on the production of offspring.

Finally, navigation underlies habitat selection, movements and resource use. The ability to navigate is critical for migration in elasmobranch species such as white sharks (*Carcharodon carcharias*) that make seasonal migrations to the `White Shark Café’ in the eastern Pacific Ocean (Boustany *et al.*, 2002; Domeier and Nasby-Lucas, 2008; Jorgensen *et al.*, 2009). Successful navigation is also crucial for species, such as lemon sharks, that return to natal areas to complete reproductive cycles in the southwestern Atlantic Ocean (Feldheim *et al.*, 2007; DiBattista *et al.*, 2008). Identification of specific cues used by elasmobranch fishes for navigation remains largely speculative. Although chemoreception has been hypothesized to facilitate navigation in teleost fishes, such as salmonids, only recently has the role of chemoreception in navigation been supported in elasmobranchs (Gardiner *et al.*, 2015; Nosal *et al.*, 2016). Evidence also supports a navigational role for the electrosensory system (Kalmijn, 1982, 2000; Anderson *et al.*, 2017; Newton and Kajiura, 2017). The possibility that elasmobranchs possess a specific sense of magnetoreception through their electrosensory system, or perhaps a separate, specific magnetoreceptive structure (e.g. iron-containing cells, magnetite or maghemite), or an optic-based cryptochrome mechanism (Anderson *et al.*, 2017) offers other avenues for linking sensory physiology to movement. Further work that investigates sensory mechanisms underlying migratory patterns is warranted and is likely to reflect evolutionary divergence based on varying life history characteristics and ecological niches across elasmobranch species (Rivera-Vicente *et al.*, 2011). Nevertheless, as navigation underlies habitat utilization, understanding the physiological and ecological factors that influence navigation could inform conservation through our understanding of habitat use and selection and what that means for interactions with fisheries.

### Neuroecology

The battery of sensory modalities described above requires a specialized and adaptable neural architecture to process and integrate information across all elasmobranch lifestyles. Relative brain size varies greatly across elasmobranch fishes and this variation has been attributed to both life history and ecology (Northcutt, 1977; Mull *et al.*, 2011; Yopak, 2012). Further, the relative size and complexity (i.e. degree of foliation) of major brain regions including the olfactory bulbs, telencephalon, diencephalon, optic tectum, tegmentum, cerebellum and medulla oblongata—termed brain organization—reflects the sensory and cognitive demands of different lifestyles and habitats (Yopak, 2012; Yopak and Lisney, 2012; Yopak *et al.*, 2014). For example, large pelagic-coastal species, such as tiger sharks (*Galeocerdo cuvier*) and white sharks, are characterized by relatively large olfactory bulbs and optic tecta, highlighting the importance of long-distance olfactory and visual cues when foraging for highly mobile and patchily distributed prey in the open ocean (Yopak *et al.*, 2014). In contrast, deep-water sharks and rays also exhibit large olfactory bulbs, but with reduced optic tecta and relatively large medulla oblongata as electro- and mechanosensory inputs are potentially more important in low light environments (Yopak, 2012).

Comparative brain morphology, specifically of the telencephalon and cerebellum, can provide clues about the cognitive ability, environment and behaviour of different elasmobranch species. The telencephalon is comprised of many subregions and nuclei responsible for the processing and integration of sensory information and has been implicated in spatial memory and sociality (Yopak, 2012). An enlarged telencephalon is characteristic of species inhabiting complex, 3D environments such as coral reef-associated sharks (*Carcharhinidae*) and those requiring integration of multiple sensory systems, such as vision, olfaction and electroreception as in hammerhead sharks (*Sphyrnidae*; Yopak *et al.*, 2007; Yopak, 2012). In contrast, the cerebellum is believed to modulate motor programmes and play a role in target tracking. As such, a large highly foliated cerebellum is characteristic of species with complex motor repertoires or prey capture modes, such as thresher sharks (*Alopiidae*), hammerhead sharks, stingrays (*Myliobatiformes*) and filter-feeding species such as whale sharks (*Rhincodon typus*; Yopak *et al.*, 2007; Yopak and Frank, 2009). These anatomical correlates can be brought to bear when developing conservation strategies for poorly understood species. Brain size and organization can provide important clues about the life history and ecology of a species, and this may be useful in mitigating ongoing or future threats, particularly for deep water or polar elasmobranchs that may be subjected to emerging fisheries in the future.

Less studied than neuroecology is the influence of neurophysiology on animal ecology. For example, skin pigmentation is altered by α-melanocyte-stimulating hormone, which is secreted from the pituitary gland, suggesting a role for brain regulation of body coloration (Visconti *et al.*, 1999). In several small-bodied elasmobranch fishes, their ability to camouflage can be modulated according to their environment, with individuals in darker surroundings becoming more pigmented than those in lighter surroundings (Gunn, 2018). In non-camouflaged scalloped hammerheads (*Sphyrna lewini*), increased pigmentation in the skin and ocular lens protect against oxidative damage from radiation in high-UV habitats (Lowe and Goodmanlowe, 1996; Nelson *et al.*, 2003). While the interaction between neuroendocrine mechanisms and environmental stimuli ultimately underlies the plasticity of body coloration, this has not been studied in detail. As a result, we argue that future studies will benefit from consideration of the physiological contribution of the brain to ecological patterns.

### Alternative tools for studying the physiological ecology of elasmobranchs

Tools are currently being developed that are specifically designed to address physiological questions in an ecological context (e.g. accelerometers and heart monitors). However, other methods can also be used to address these types of questions. Here, we highlight the utility of both novel and traditional techniques that provide alternative perspectives for investigating the physiological ecology of elasmobranch fishes.

#### Genomics

Over the past three decades, molecular genetic tools have rapidly advanced and have increased the amount of data that can be obtained from an individual sample. In particular, the development of high-throughput sequencing (HTS) technologies and associated bioinformatic analyses has increased the capacity of genetic tools to answer a wide variety of physiological and ecological questions (e.g. phylogenomics, metagenomics/barcoding and functional genomics; Corlett, 2017; Komoroske *et al.*, 2017). These advances and their applications could provide a better understanding of the relationship of poorly understood species to their environments and ecological communities. Moreover, HTS-based genomic analyses provide an opportunity to understand the molecular mechanisms that drive many of the established ecophysiological patterns documented in this taxon.

Phylogenomics, using HTS approaches, has enabled a more robust understanding of evolutionary relationships (Lemmon and Lemmon, 2013), by allowing for the inclusion of more individuals and loci in phylogenetic analyses. Since physiology is a product of a taxa’s evolutionary history, it is important to consider the underlying evolutionary trajectory of a lineage and their relationships with other taxa when studying physiological ecology across a diverse clade such as elasmobranchs (Garland and Carter, 1994; Cooke *et al.*, 2014; Stein *et al.*, 2018). For example, understanding the morphology and function of elasmobranch brains requires accounting for phylogeny (i.e. evolutionary non-independence) to properly assess the effects of life history and the environment on neurobiology (Mull *et al.*, 2011). Additionally, approaching questions from a phylogenetic framework can highlight evolutionary innovations in distantly related lineages—such as filter feeding, a trait that has multiple, independent derivations within elasmobranchs—which can, in turn, help explain the mechanisms behind the trait (Martin and Naylor, 1997). Recently, novel comparative methods have been developed to examine correlated evolution between species’ traits and environmental parameters (threshold models), to examine the effect of traits on the dynamics of speciation and extinction (state-dependent speciation and extinction) and to elucidate the drivers of trait evolution (phylogenetic path analysis; FitzJohn, 2012; von Hardenberg and Gonzalez-Voyer, 2012; Revell, 2013). Thus, genomics clearly compliments our understanding of the contemporary physiological ecology of elasmobranch fishes by providing an evolutionary perspective.

Aspects of environmental habitat use and quality, previously only measured as abiotic parameters (e.g. temperature, salinity and dissolved oxygen), can now be inferred through metagenomics/barcoding. For example, microbial communities living within or on the bodies of elasmobranch fishes can be assessed through HTS shotgun sequencing, with the added capability to also assess individual and population health and environmental condition (Doane *et al.*, 2017). Environmental DNA analyses (e.g. species-specific primer and targeted gene sequencing approaches) may have the potential to efficiently identify which elasmobranch species inhabit different habitats (Sigsgaard *et al.*, 2016; Simpfendorfer *et al.*, 2016; Weltz *et al.*, 2017). These results, in turn, can provide insight into the environmental conditions under which certain species can be found, which may elucidate some of their physiological tolerances.

Functional genomics, which uses mRNA sequencing to identify the instantaneous expression of genes in particular tissues (also known as the transcriptome), is an exciting application of HTS genomic tools. Since the regulation of gene expression is one of the primary cellular mechanisms governing metabolism and physiological processes, functional genomics will aid our understanding of how physiology relates to ecology in elasmobranch fishes. Functional genomics has been used to determine the molecular mechanisms behind endothermy (Richards *et al.*, 2013), immune response (Goshima *et al.*, 2016; Hsu, 2016), reproduction (Swift *et al.*, 2016) and brain development (Pose-Méndez *et al.*, 2016). Additionally, the application of epigenetic tools can be used to research the mechanisms of adaptive responses that span generations, which is becoming a major concern as elasmobranch fishes respond to the effects of fishing (Stevens *et al.*, 2000; Frisk *et al.*, 2005) and climate change (Lighten *et al.*, 2016; Peat *et al.*, 2017).

#### Chemical tools

Stable isotope analysis (SIA) has traditionally been viewed exclusively as an ecological tool, due to its past utility to study trophic ecology and the movement of marine species (Hobson, 1999; Graham *et al.*, 2010; Carlisle *et al.*, 2012; Hussey *et al.*, 2012). However, SIA exploits the natural integration of the external chemical environment into organismal tissues, through feeding, which is typically governed by physiological process. As SIA has become more prominent, researchers increasingly recognize the need for understanding how physiological mechanisms ultimately influence the isotopic composition of an organism’s tissues to ensure that results of SIA can be interpreted in a meaningful way for ecological studies (Martínez del Rio *et al.*, 2009; Hussey *et al.*, 2012). The isotopic composition of tissues is dictated by the physical environment of an organism (e.g. salinity, temperature and dissolved oxygen; Kalish, 1991; Mohan and Walther, 2016; Mont’Alverne *et al.*, 2016), but it is increasingly recognized that biological variables (e.g. rates of growth or feeding) play a role as well. Thus, understanding the physiological mechanisms underpinning stable isotope dynamics is inherently necessary for accurate ecological interpretation. For instance, the isotopic composition (δ^13^C, δ^15^N) of a tissue is affected by tissue break down and regrowth (e.g. tissue turnover), which in turn is influenced by an individual’s metabolism and/or growth at particular life stages that governs how quickly ingested prey items are assimilated and converted into tissue biomass (Tieszen *et al.*, 1983; Logan and Lutcavage, 2010; Vander Zanden *et al.*, 2015). Since different tissues turnover at different rates, selection of a particular tissue for study must be done with care to ensure the chemical data obtained are correctly interpreted (Carlisle *et al.*, 2016; Li *et al.*, 2016). In addition, an understanding of diet–tissue discrimination factors (Hussey *et al.*, 2010), differences in isotopic composition between an animal and prey (McCutchan *et al.*, 2003) and diet quality (Caut *et al.*, 2009) is also necessary to characterize trophic relationships among predators and prey. Considering the important influence of physiology on stable isotope dynamics, which has implications for ecological interpretations, the power of SIA as a tool is greatest when considered through the lens of physiological ecology.

Unlike SIA, ecotoxicology is not often considered in the context of physiological ecology; however, aspects of this field require the integration of both ecological and physiological parameters, lending itself useful as a tool for this discipline. The sources of contaminants of interest are usually anthropogenically derived (e.g. organochlorines) or influenced by anthropogenic activities (e.g. mining that releases trace/heavy metals). Unlike stable isotopes, which follow predictable patterns (Peterson and Fry, 1987) through food webs (i.e. nitrogen), with productivity (i.e. carbon) or with temperature (i.e. oxygen), contaminant accumulation is more dynamic. Factors such as the history of contaminant release, time since release and magnitude of release will influence the contaminant signatures of geographic regions. Similar to stable isotopes, contaminants are acquired primarily through ingestion (Gobas *et al.*, 1999). Thus, animals must be interacting with their environment to acquire these contaminant signals. This concept has been applied to study ecology of migratory animals such as humpback whales (*Megaptera novaeangliae*; Elfes *et al.*, 2010) and albacore and bluefin tuna (*Thunnus alalunga, Thunnus thynnus*; Dickhut *et al.*, 2009; Chouvelon *et al.*, 2017). In these cases, researchers compared contaminant signals for various groups of animals to make inferences on the core areas where these animals may be feeding. Elasmobranch fishes exhibit characteristics that make them amenable for using contaminant markers to study their ecology. Many species migrate (Weng *et al.*, 2008; Jorgensen *et al.*, 2009; Bansemer and Bennett, 2011) and typically occupy upper trophic levels, both of which make them prone to accumulate contaminants (Fisk *et al.*, 2002; Storelli *et al.*, 2005; Silva *et al.*, 2009; Lyons *et al.*, 2013).

While contaminant signatures can be used as ecological markers, physiology also plays a role in influencing contaminant accumulation. Not only does the location of feeding (ecology) influence contaminant uptake, but physiological factors also dictate feeding rate. For example, elasmobranch fishes with higher aerobic metabolic rates tend to have higher concentrations of organic contaminants (Lyons *et al*. *in review*). This results from either higher rates of feeding, feeding on more calorically dense prey items, or both. Other physiological factors include sex differences. Females offload contaminants to their young (Lyons and Lowe, 2013; Lyons and Adams, 2015), enabling them more opportunities to reduce contaminant concentrations in their tissues compared to males. The degree to which females may offload contaminants therefore likely results from an interaction of reproductive physiology and ecology (Lyons *et al*. *in review*).

Mercury is unique from other anthropogenic chemicals in that it occurs naturally, although concentrations are increasing due to human activities (Pacyna *et al.*, 2010). Mercury concentrations in tissues are influenced by both ecological and physiological factors, and these can interact to affect accumulation. For example, McKinney *et al.* (2016) found both growth rate (using total length as a proxy for age) and trophic position influenced mercury bioaccumulation in 17 shark species. In other cases, ecological variables (e.g. foraging depth and habitat type) do not always accurately predict mercury accumulation, suggesting an influence of multiple factors. Foraging depth is a significant factor in mercury concentrations for pelagic teleosts and their prey (Choy *et al.*, 2009), and higher concentrations in crocodile sharks (*Pseudocarcharias kamoharai*) compared to other pelagic shark species were attributed to its feeding in the deeper mesopelagic food web (Kiszka *et al.*, 2015). However, Pethybridge *et al.* (2010) found results conflicting with the foraging depth hypothesis with higher concentrations in squalids inhabiting the continental shelf than in slope species. Exploring the suite of physiological (e.g. growth rate) and ecological factors that influence contaminant accumulation can benefit conservation efforts by indicating species or habitats that are more susceptible to contaminant accumulation, and by extension, contaminant effects.

## Future directions

There is a need for the field of physiological ecology to develop a framework to generate outputs that are accessible and relevant to conservation and management. For example, understanding physiology is critical to mitigating threats of overexploitation because it can dictate organismal responses to fishing practices (e.g. at-vessel and post-release mortality). Detrimental effects of these responses can be mitigated through modifications of fishing practices, such as electromagnetic deterrents that limit interactions of sharks with pelagic longline gear (Brill *et al.*, 2009; Hart and Collin, 2014). Without the ability to translate physiological ecology to management practices, integrative approaches that strengthen this field will not result in realized conservation changes. Thus, collaboration among physiological ecologists with resource managers, policy makers and stakeholders is crucial for appropriate implementation of effective management plans at the population level.

In addition to fishing pressure, climate change presents a challenge for many species as niches of both prey and predators are likely to shift geographically according to species-specific physiological abilities, habitat preferences and metabolic demands. Increasing water temperatures are predicted to increase metabolic rates (Rosa *et al.*, 2014; Pistevos *et al.*, 2015), which begs the question as to whether animal’s body plans (e.g. gill and heart morphology) are equipped to match the physical demands imposed by climate change and associated modification of the habitats for which they were originally adapted. Exposure to anthropogenic contaminants also poses threats to the performance and health of elasmobranch fishes in unexpected ways. For example, the olfactory sensitivity of Atlantic stingray to amino acids is reduced after acute exposure to mixtures of crude oil, which may impair their ability to forage successfully (Cave and Kajiura, 2018).

Finally, ecological and conservation field-based research on elasmobranch fishes tends to focus on the largest and most charismatic species (e.g. white sharks and *Mobula* spp.) even though they may not face the highest risk of extinction or be the best candidates to answer particular research questions. Therefore, we argue that future work must carefully select study species and develop appropriate model organisms; it is our hope that the Physiological Ecology Symposium at the 2017 Annual Meeting of the American Elasmobranch Society and this *Perspective* spur continued discussion between ecologists and physiologists as to how to integrate their respective disciplines to enhance our understanding of the biology of sharks, skates and rays as well as to improve conservation efforts for many members of this group of vertebrates that are simultaneously threatened and understudied species.
